# Dental Management of Patients With Amyotrophic Lateral Sclerosis

**DOI:** 10.7759/cureus.50602

**Published:** 2023-12-15

**Authors:** Nasser AlMadan, Ali AlMajed, Mohammed AlAbbad, Fadhel AlNashmi, Abdulmohsen Aleissa

**Affiliations:** 1 Dental Department, Ministry of Health, Riyadh, SAU

**Keywords:** hospital dentistry, special care dentistry, dental management, als, amyotrophic lateral sclerosis

## Abstract

Amyotrophic lateral sclerosis (ALS) is a neurodegenerative disease that affects the upper and lower motor neurons with upper and lower motor neuron manifestations. It is divided into two variants: a spinal onset and a bulbar onset. The first starts as focal muscle weakness and wasting that spreads with disease progression, while the second phenotype presents with dysarthria, dysphonia, and dysphagia. Moreover, an extra-motor manifestation could be reported with the most commonly reported symptoms being the change in cognition and sleep disorder. Oral manifestations include increased salivation, limited mouth opening, and dysphagia. Patients with ALS have difficulty maintaining oral hygiene, and it is important for the practitioner and the caregiver to take care of this group of population. We herein provide a short review of the disease with a focus on the oral manifestations and dental considerations for management for this group.

## Introduction and background

Amyotrophic lateral sclerosis (ALS) is a neurodegenerative disease that affects the motor neurons with upper and lower motor neuron manifestations, in addition to extra-motor manifestations. It is divided into two phenotypes: a spinal type that usually starts as focal muscle weakness and wasting that spreads with disease progression, and a bulbar phenotype that presents with dysarthria, dysphonia, and dysphagia. The most commonly reported extra-motor manifestation is a change in cognition, sleep disorders, autonomic disturbance, and skin elasticity loss. ALS is divided into sporadic cases, which account for most of the cases, and familial cases. ALS typically manifests in the old population, with familial cases having a lower age of onset [1-4]. Oral manifestations reported in ALS include increased salivation, trismus, and dysphagia, all of which have implications for maintaining good oral hygiene and providing dental care for this group of individuals. The average annual incidence of ALS varies, with most studies reporting higher incidence in men than women, with a peak incidence at 70-79 years of age. Wolfson et al. reported that incidence ranges across different countries. It is estimated to be 0.26 per 100,000 in Ecuador to 23.46 per 100,000 in Japan. Prevalence also ranged from 1.57 per 100,000 in Iran to 11.80 per 100,000 in the United States of America [5]. Age of onset varies depending on the type with sporadic cases manifesting in the sixth to seventh decade, while the familial form occurs at a younger age. Males have a higher risk of developing sporadic limb onset ALS compared to females [1]. This short review will briefly discuss ALS, emphasizing oral manifestations and dental management considerations.

## Review

Pathophysiology and etiology

ALS is a progressive neurodegenerative disorder that is caused by the interplay of a myriad of epigenetic, environmental factors, and genetic factors with more than 20 identified genes to date [1]. The neurophysiological techniques identified the cortical hyperexcitability in the disease pathogenesis, and it could be used as a novel diagnostic marker as it can differentiate ALS reliably from the mimickers. ALS is characterized by neuromuscular connection loss, axonal retraction, upper motor neurons, and lower motor neuron cell death [1]. Dysfunction of the astrocytic excitatory amino acid transporter 2 (EAAT2) leads to reduced glutamate uptake from the synaptic cleft, leading to glutamate excitotoxicity leading to neurodegeneration through activation of calcium-dependent enzymatic pathways. It increased oxidative stress due to the superoxide dismutase-1 (SOD-1) gene mutation, which induced mitochondrial dysfunction and defective axonal transportation [6,7].

Clinical features

It is divided into two phenotypes: a limb-onset ALS with upper and lower motor neuron signs in the limb and a bulbar onset ALS characterized by speech and swallowing difficulties, followed by limb weakness in the late stages [2]. Limb onset is characterized by progressive muscle weakness with a focal onset that spreads to adjacent body regions. It usually starts in the limb muscles, most affecting the distal muscles first with the patient perceiving a slight weakness in the distal part of the limb that progresses and spreads to the adjacent part of the affected limb. Subsequently, the disease progresses to the opposite limb. Muscle atrophy, muscle cramps, and stiffness accompany the weakness [1]. Bulbar onset is characterized by dysarthria and dysphagia, followed by limb weakness. Neurological disorders are very common in this group of patients, with depression, dementia, Parkinson’s, and epilepsy found more commonly than the normal population [2]. Sialorrhea is seen in the bulbar-onset ALS due to difficulty swallowing saliva and weakness of the facial muscles from the upper motor neuron damage, which leads to difficulty maintaining the lip seal and blow cheek [1,3].

Medical management

Management of ALS is mainly symptomatic, with multiple caregivers involved. Patients with ALS patients commonly suffer from chronic respiratory failure due to weakness of the diaphragmatic and intercostal muscles. It is managed initially with chest physiotherapy and frequent suctioning. As weakness progresses, tracheostomy, chronic ventilatory support, and noninvasive positive pressure ventilation are employed in management [3]. Masticatory and swallowing muscle weakness leads to dysphagia that can result in weight loss. In the early stages, dysphagia is managed with diet modifications and safe swallowing techniques; however, at late stages, with the increased risk of aspiration, enteral nutrition is considered [3]. Dysarthria has no active cure with little benefits gained from speech therapy, and this can be frustrating for the patient. It is usually managed with symptomatic and compensatory strategies that can help communicate and improve patients' quality of life. The patient can move from oral to written communication, use an augmentative communication device, or via another person [8,9]. Painful muscle spasm is managed with mexiletine or levetiracetam [10]. Botulinum toxin injections into the spastic muscles can be used if oral therapy is not effective [3]. Regarding sialorrhea, it is managed with anticholinergic medication, including amitriptyline and glycopyrronium bromide [2]. One study assessed the effectiveness of radiotherapy for sialorrhea and found a reduction in sialorrhea in 78.6% of patients with ALS who failed pharmacological agents [11]. A recent Cochrane review concluded that there is a low-certainty to moderate-certainty evidence for the use of botulinum toxin B injections to salivary glands and moderate-certainty evidence for the use of oral dextromethorphan with quinidine for the treatment of sialorrhea in motor neuron disease [12]. Physical therapy could help in slowing down neuromuscular degeneration and improve daily activity for ALS patients. Patients also should be provided with assistive devices as the disease progresses, including neck collars, ankle foot orthosis, canes, crutches, and a wheelchair [2,3]. Riluzole remains the only approved disease‐modifying drug. It has anti-glutamatergic effects and prolongs the mean patient survival by three to six months with the most commonly reported side effects, including nausea, diarrhea, fatigue, dizziness, and liver problems. More recently, the free radical scavenger edaravone has been used for ALS with promising results [1].

Dental management

A dental practitioner has to develop a communication method with the patient, as patients with advanced disease cannot communicate well. This can be solved via the caregiver, written communication, or external augmentation devices. Patients with advanced disease cannot perform oral hygiene, and a nursing staff or a guardian usually carries it out. A cheek retractor could be helpful in order to facilitate accessibility to teeth and mouth during oral hygiene practice. Stabilization of the lower jaw by a dental shield is recommended for bite support to relieve the fatigue of the jaw muscles and to prevent biting on the caregiver's fingers. A tongue scraper is recommended to remove the excess debris and to manage the coated tongue. Chlorhexidine mouthwash is also recommended to reduce bacterial load and prevent periodontal disease. Dental management should be carried out with a soft cushion to minimize the pressure applied on the back and help obtain a relaxed position during dental treatment.

Additionally, a mouth gag can aid in cleaning and during dental treatment to facilitate mouth opening and provide access during dental work [13,14]. A study in the Netherlands found that most patients with ALS were not satisfied with their daily oral care [15]. Thus, the caregiver and the clinician must discuss the best possible strategies for dental care. A rubber dam and high-volume suction and saliva ejectors are necessary for dental treatment, as this cohort of patients has difficulty swallowing and increased salivation. It is also possible to give anticholinergic medication to reduce salivation. As the patients have limited mouth openings, a mini-head dental handpiece helps provide dental treatment in the posterior teeth. Patients are instructed to apply the mouth-opening exercise regimen, physiotherapy, and TheraBite jaw motion rehabilitation system [16].

Oral manifestations

Oral manifestations for ALS patients include sialorrhea, and it is seen predominantly in patients with a bulbar form of the disease. It could be related to tongue spasticity, weakness in facial muscles, and buccal incompetence of buccal muscles. Another salivary complaint is the retention of thick, viscous saliva. The risk of hypersalivation includes the development of angular cheilitis, difficulty in speaking, sleep disturbance, and increased risk for aspiration. The combination of increased saliva and weakness of the tongue and respiratory muscles could lead to aspiration pneumonia. Interestingly, patients with sialorrhea were associated with poor oral status, amount of tongue coating, and increased gingival inflammation. However, another study concluded that increased salivation was associated with lower gingival inflammation and less risk of dental caries, and it was attributed to the buffering and bactericidal effect of saliva [16]. Muscle weakness can result in dysphagia that leads to food debris staying in the mouth, which subsequently leads to the promotion of periodontal diseases [17-19]. Additionally, many reports described cases with macroglossia, atrophic tongue, fasciculation in the tongue, masticatory muscle pain, and progressive limitation of mouth opening [14,17,20]. Macroglossia tends to increase in prevalence with the progression of the disease, and it has a negative impact on communication [21-23]. Maximum mouth opening is reduced in patients with ALS. This could result in difficulty in maintaining oral hygiene and providing dental treatment.

Recommendations

(1) The dental team has to discuss oral hygiene strategies that fit the patient the most with the patient and the caregiver.

(2) Jaw exercises must be implemented to reduce the progression of limited mouth opening.

(3) The patient has to be placed on a strict follow-up protocol to mitigate the dental complications as early as possible.

## Conclusions

This study covered a broad range of information about amyotrophic sclerosis and the challenges they face in maintaining good oral health. It is also very challenging for the dental practitioner to deal with the complications associated with this disease. A collaboration between the dental team and caregivers is important to maintain adequate oral hygiene and reduce the chances of developing dental complications.
